# Pulsation of electrified jet in capillary microfluidics

**DOI:** 10.1038/s41598-017-05477-9

**Published:** 2017-07-18

**Authors:** Xiong Li, Shanshan Wei, Liucheng Chen, Gang Qu, Huisheng Zhang, Zhou Liu, Liqiu Wang, Tiantian Kong, Tianfu Wang

**Affiliations:** 1 Guangdong Key Laboratory for Biomedical Measurements and Ultrasound Imaging, Department of Biomedical Engineering, School of Medicine, Shenzhen University, Shenzhen, China; 2 College of Chemistry and Environmental Engineering, Shenzhen University, Shenzhen, China; 3 Department of Mechanical Engineering, The University of Hong Kong, Hong Kong, China; 4 HKU-Zhejiang Institute of Research and Innovation (HKU-ZIRI), Hangzhou, Zhejiang China

## Abstract

In this work, we investigate the pulsation of an electrically charged jet surrounded by an immiscible dielectric liquid in flow-focusing capillary microfluidics. We have characterized a low-frequency large-amplitude pulsation and a high-frequency small-amplitude pulsation, respectively. The former, due to the unbalanced charge and fluid transportation is responsible for generating droplets with a broad size distribution. The latter is intrinsic and produces droplets with a relatively narrow size distribution. Moreover, the average size of the final droplets can be tuned via the intrinsic pulsating frequency through changing the diameter of the emitted liquid jet. Our results provide degree of control over the emulsion droplets with submicron sizes generated in microfluidic-electrospray platform.

## Introduction

Emulsions are liquid droplets dispensed in another immiscible liquid phase^1^. They are useful for a myriad of applications, particularly when the droplets are fine in size^2–7^. Many pharmaceutical and cosmetic products are emulsion-based^8, 9^, and emulsion droplets have been used for single-cell barcoding and sequencing^10–12^, for molecular imaging^3, 4, 6, 13^ and tumor-targeting photo-acoustic therapies^14, 15^. For most therapeutic applications, the emulsions are expected to have high loading efficiency, and thermodynamically stable with a narrow size distribution. The droplet size is preferably in nanometer range to facilitate cellular uptake, and prevent clogging the blood vessels^2, 6, 7^.

Emulsification approaches are plentiful, the “top-down” category mostly involves mixing two immiscible liquids in bulk processes such as homogenizing and shaking, while the “bottom-up” category is microfluidic-based to generate emulsions at the level of individual droplets^16–26^. The former produces nano-sized emulsion droplets with a broad distribution in large-scale, but little control over the formation of individual droplets is available. Moreover, the “over-processing” is often observed in high-pressure emulsification, in which a higher-energy input leads to a larger average droplet size^27^. This is due to the “re-coalescence” between the newly created droplet and surrounding ones. The microfluidic method generates uniform and well-defined microdroplets discretely^18, 28^. The generated droplets normally have sizes on the scale of the diameter of the injection tip. Due to the high driven pressure at micro-channels, the size of the generated microdroplets is limited to 10 micrometers and is difficult to reduce further^29^. Thus, the method to generate nano-sized emulsion with control over individual droplets is highly desired.

Electro-spraying a liquid phase in a surrounding immiscible liquid bath is known to produce nano-emulsions with reasonable polydispersity^30–32^. The polydispersity in droplet size for electrospray-in-bath is typically larger than that for electrospray-in-air. This is due to fact that the charged droplets cannot be transported away from where they are generated^33^. They either re-coalesce with new droplets, or interfere the local electric field, leading to the generation of droplets with uneven sizes. Microfluidic-electrospray is a technique where the electrosprayed droplets are flushed away by a flow-focused immiscible phase on microfluidic chips^29, 33^. It has been demonstrated to offer nano-sized emulsions with a narrow size distribution, but only with a few combinations of imposing flow rates and applied electric field strength^29, 33^. The operation modes in microfluidic-electrospray approach have not been sufficiently characterized, and the process capability of this technique is inadequately explored. Thus, the degree of control over droplet size and size distribution is not yet clear to the end-users yet.

In this work, we systematically study the operation modes of the electrically emitted droplets in immiscible liquids using a flow-focusing capillary microfluidic device. We report two pulsating modes of the charged liquid with distinctive frequency and amplitude respectively. In the low-frequency pulsation, the liquid meniscus pulsates between a sharp and relaxed conic shape with a large amplitude. As such, small and large droplets are generated alternatively, and the resultant droplets have a broad size distribution. In the high-frequency pulsation, the cone-jet pulsates between different cone angles periodically with a small amplitude, generating small droplets with a narrow size distribution. Moreover, by increasing this intrinsic pulsating frequency, we can tune the average droplet size to achieve control over the resultant emulsions. Our results provide guidelines for generating droplets with degree of controlled over their size and distributions in microfluidic-electrospray platform.

## Results

**Figure 1:**
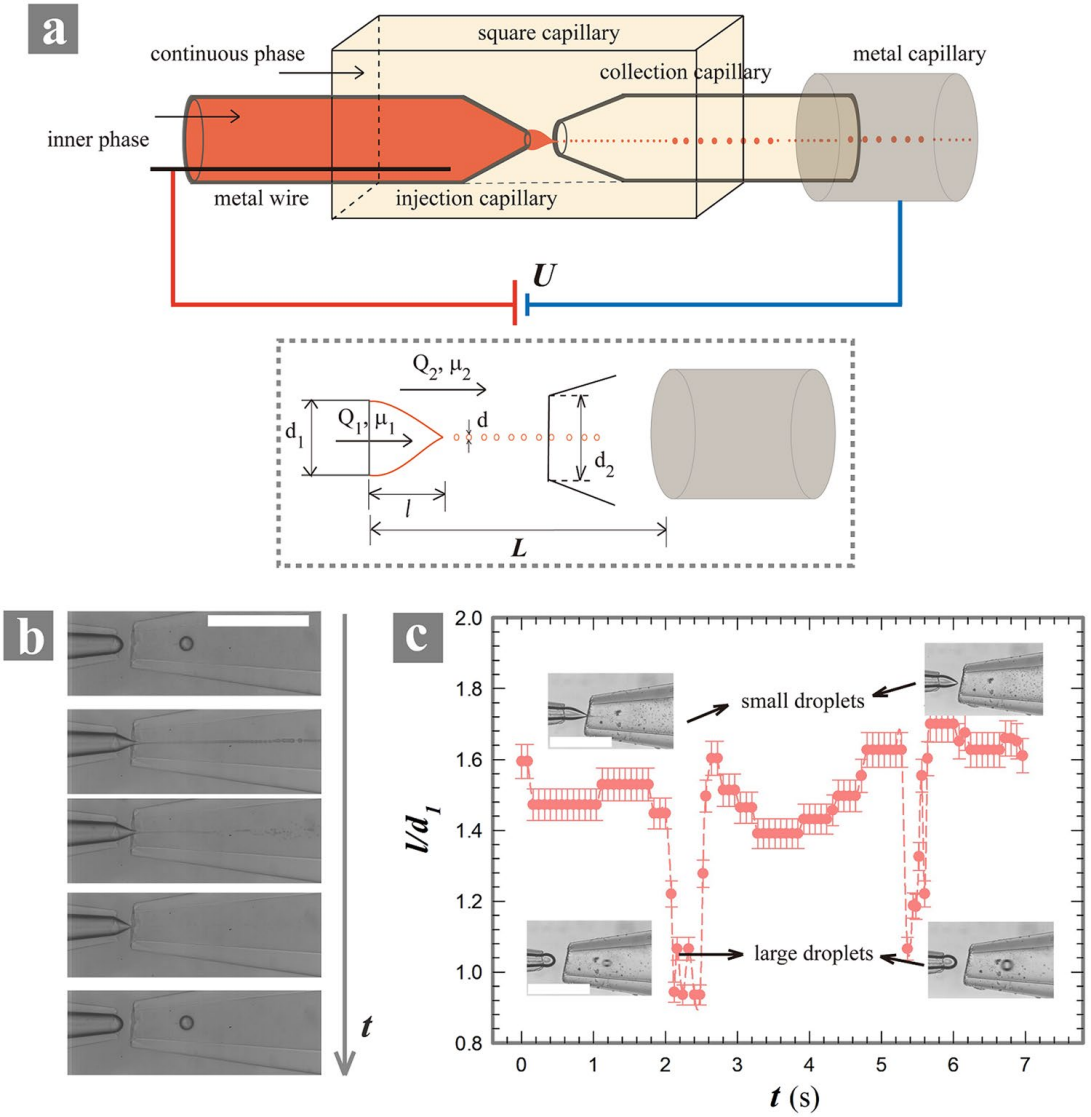
(**a**) Schematic of the experimental setup (up) the physical parameters of the system (bottom); (**b**) Series of optic microscopic images showing a pulsation cycle of droplet generation under the same applied voltage, 1 kV. The scale bar is 1 mm. The inner and outer phases are deionized water and paraffin oil with 5 wt% span 80, respectively. The inner and outer flow rates are 5 μl/hr and 2000 μl/hr, respectively; (**c**) The alternative generation of small and large droplets due to the pulsating tip of the inner phase. The time interval is 8 mili-seconds. The scale bar is 500 µm.

We fabricated the capillary microfluidic device by aligning two tapered round capillaries, an injection and a collection, in a square capillary^18^, as shown schematically in Fig. 1a. The diameters of the injection and collection capillaries were denoted as *d*_*1*_ and *d*_*2*_ respectively. Deionized water was used as the inner phase that flowed through the injection capillary at a flow rate of *Q*_*1*_ driven by a syringe pump (Longer Pump). We used dielectric oils, including paraffin, silicone oil or squalene as the outer phase. Surfactants, span 80 (Sigma) or ABIL EM 90 (Evonik), was added in the outer phase to modulate the interfacial tension and to stabilize the generated droplets. The inner aqueous phase was charged positively by a direct-current (DC) high voltage supply through an inserted electrode in the injection capillary. The outlet of the device was connected to ground through a metal capillary, as shown schematically in Fig. 1a. The DC voltage was applied between the injection and metal capillaries to create an electric field that induced an electrohydrodynamic flow. The resultant electric field strength *E* was estimated as *E* = *U/L*, with *U* being the potential and *L* being the distance between the water tip and the ground electrode. We focused on the dynamic behaviors of the charged liquid meniscus under a DC-applied voltage. The charged liquid were visualized using a microscope (Motic AE2000) coupled with a high-speed camera (Phantom M110). Due to the tiny size of the generated droplets in electro-microfluidics, measurement of the their diameter through high-speed images has little accuracy. Given the fact that the water tip would pulsate after emitting each droplet, we therefore characterize the generation of droplets by characterizing the pulsation of the water tip. The length of the water tip *l* as well as the pulsating frequency *f* were measured from the high-speed images.

Without the applied voltage, the tip of the inner phase is hemispherical and the generated droplets are uniform in dripping regime. When a voltage is applied, the inner phase is charged and the meniscus is stretched into a conical shape (Fig. 1b). At the end of the cone, a thin jet emits and breaks up into tiny droplets of sub-micron sizes. However, this process only maintains for a certain period of time, and the sharp cone would gradually relax back to a hemispherical one which emit much larger droplets (Fig. 1b). Afterwards, the hemispherical interface is recharged and becomes cone-shaped again, following which submicron droplets are produced. The transition between the cone and the hemispherical shapes of water tip is periodic, as shown in Fig. 1c. During the transition between these two states, droplets with intermediate sizes are generated (Video S1).

**Figure 2:**
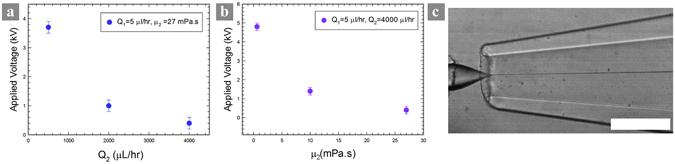
The onset applied voltage at which a sharp cone forms decreases with an increasing (**a**) flow rate or (**b**) viscosity of the outer phase; In plot (**a**,**b**), all the other parameters are kept constant, and the outer phases with different viscosities are hexadecane (1.5 mPa.s), silicone oil (10 mPa.s) and squalene (27.8 mPa.s), respectively. (**c**) With sufficiently large shear stress from the outer phase to overcome capillary pressure, a sharp cone is observed without applied electric voltage. The outer phases are paraffin oil (40 mPa.s) with 5% EM 90 with a flow rate of 2000 μl/hr. The scale bar is 200 µm.

In microfluidic-electrospray, the meniscus is mainly subjected to three stresses: the electrostatic stress exerted on the meniscus surface, the shear stress from the surrounding liquid phase, and the surface tension. The former two promote an elongated jet while the latter tries to maintain a hemispherical shape. As the sum of $\frac{1}{2}{\varepsilon }_{0}{E}^{2}\,$and ${\mu }_{2}\frac{{Q}_{2}}{{d}_{2}^{2}{d}_{1}}$ exceeds the capillary pressure 4*γ*/*d*_1_, the sharp cone forms, where *μ*_2_ are the viscosity of the surrounding oil phase; *γ* is the interfacial tension between the water and oil; *ε*_0_ is the permittivity of the free space. As a result, a higher *Q*_2_ or *μ*_2_ leads to a lower applied *E* to form a cone-shaped jet (Fig. 2a,b). At a sufficiently large *Q*_2_ and *μ*_2_, the tip is conical without any applied electric field (Fig. 2c). This requires a high pumping power that could be a technical problem for less-robust microfluidic devices.

**Figure 3:**
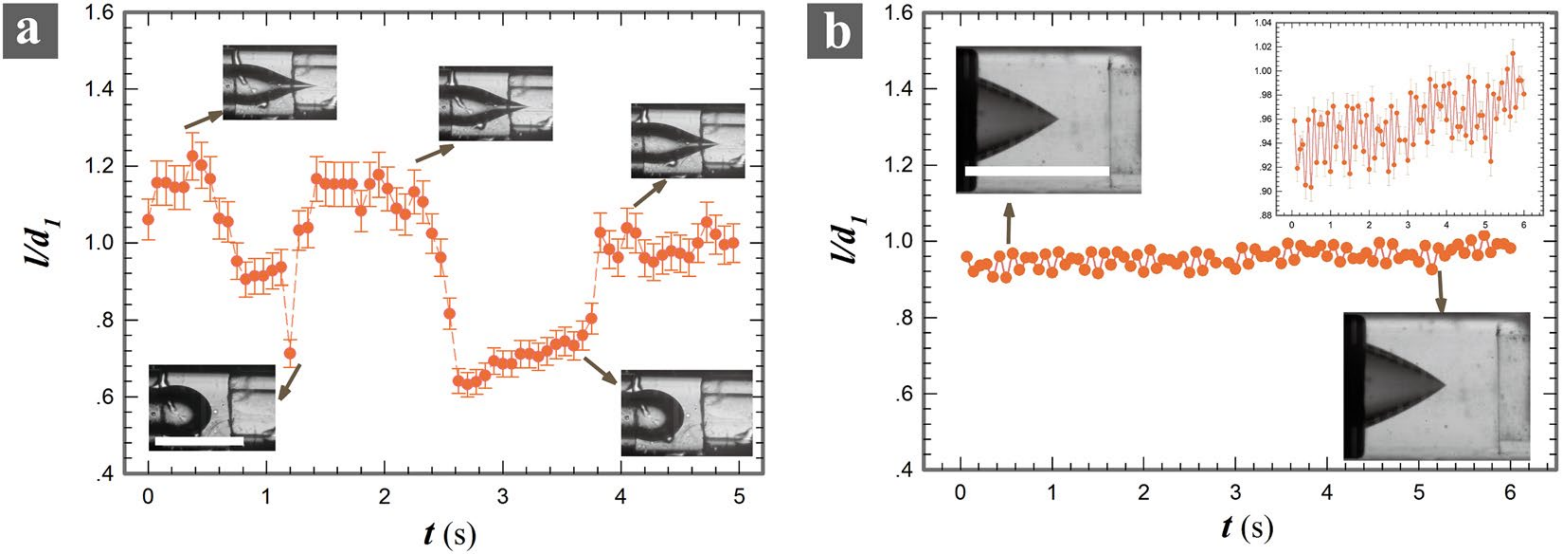
A plot of the normalized length of the water tip against time under a constant applied voltage U = 11 kV at different flow rates: (**a**) *Q*_*1*_ = 5 μl/hr and *Q*_*2*_ = 6000 μl/hr; (**b**) *Q*_*1*_ = 60 μl/hr and *Q*_*2*_ = 6000 μl/hr. The analyzed images are extracted every 30 frames from a high-speed video recorded at 400 fps. Scale bars are 1 mm. (Inset: close-up of the plot (**b**).

The charged jet pulsates periodically in two distinctive modes with a low and high frequency, ~1 Hz and ~10^2^ Hz, respectively^34, 35^. In the low-frequency mode, the cone-shaped jet sprays for approximately 1 second, and then relaxes to generate large droplets for also 1 second. Subsequently the hemispherical tip becomes conical and a new cycle begins. The pulsating amplitude, represented by the tip length *l* of the meniscus, at low-frequency mode is at the same scale of the nozzle diameter *d*_*1*_ (Fig. 3a). The droplets collected in this mode have a large polydispersity since both small and large droplets are produced alternatively.

The low-frequency pulsation occurs when the imposed electric stress is not comparable to the shear stress by the outer fluid. The large shear stress leads to the droplet detachment from the injection nozzle. After the droplet detaching, the cone volume becomes small and thus the curvature is large (Fig. 3a, inset). The increased curvature increases the capillary pressure to dominate over the shear stresses, therefore the meniscus could keep a hemispherical shape. Gradually with the imposing flow rate, the volume builds up and the curvature decreases to a point that the capillary pressure is overcame again. Then the conical tip forms and emits tiny droplets until a new cycle. As a result, the low-frequency pulsation can be suppressed by decreasing the velocity difference between the inner and outer fluids. Indeed, as we increase *Q*_*1*_, the conical tip lasts for tens of minutes during our observation period, as demonstrated in Fig. 3b and Video S2. This indicates that a delicate balance between charge and mass transportation must be provided for a stable droplet generation.

**Figure 4:**
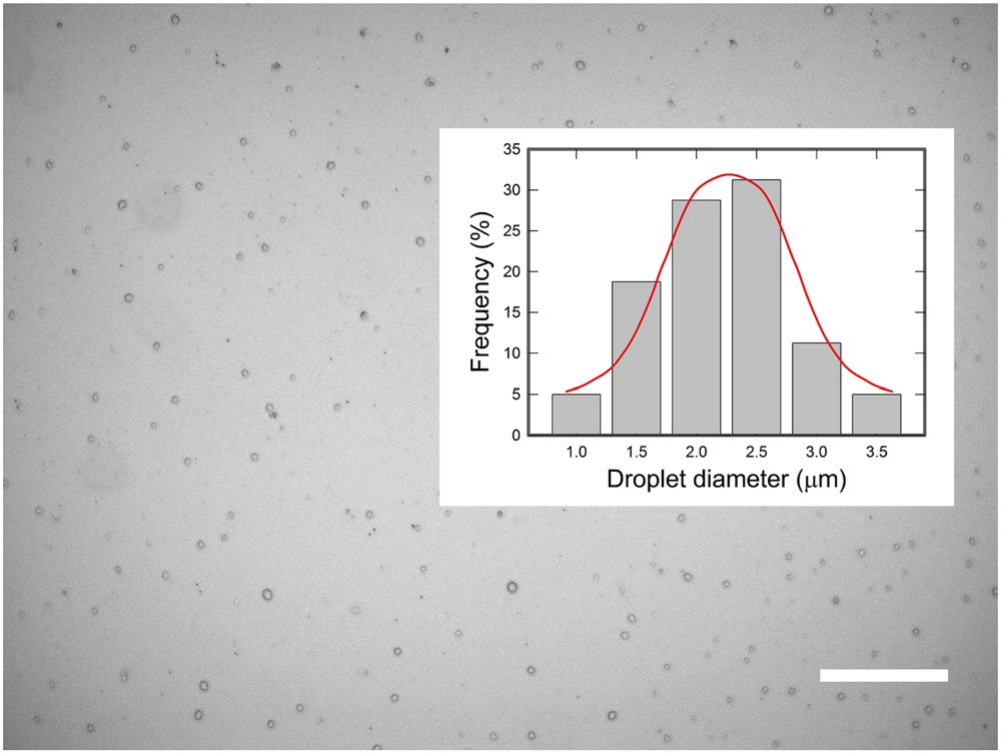
Drops of DI water in paraffin oil obtained in the high-frequency pulsation mode. The scale bar is 50 µm (Inset: size distribution).

**Figure 5:**
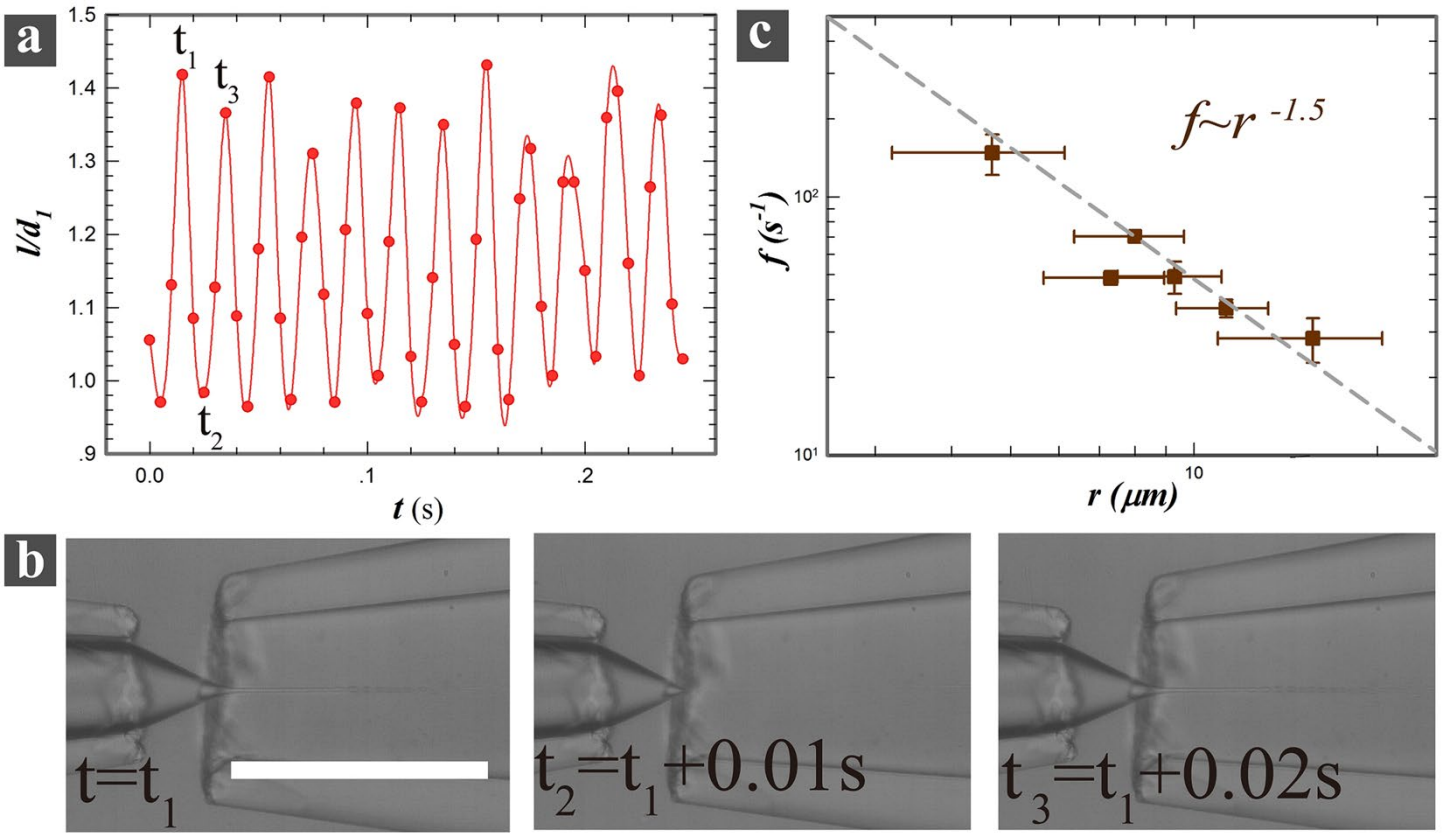
(**a**) A plot of the length of the water tip against time under a constant applied voltage in the high-frequency pulsating regime. The analyzed images are extracted every 2 frames from a high-speed video recorded at 1000 fps; (**b**) Representative images corresponds to a cycle of pulsation at a frequency of 50 Hz. The scale bar is 500 µm; (**c**) The dependence of pulsating frequency *f* on the emitted jet size, *r*, indicating a power-law fit with an exponent of −1.5.

As the shear stress is comparable to the applied electric stress, the high-frequency pulsation starts where the conical tip oscillates between different angles (Fig. 3b, inset plot). The droplets collected in this mode have a much narrower size distribution (Fig. 4) than that in low-frequency mode. The size distribution is Gaussian and has an average droplet size of 2.25 µm and a width of 1.2 µm, as shown in the inset of Fig. 4. According to mass conservation, under constant volumetric flow rate, the faster droplets are generated, the finer they should be. Thus, the average droplet size can be influenced if the intrinsic pulsating frequency can be tuned. The pulsating frequency reported for electrospray-in-air has a magnitude around 1 kHz, while that in flow-focusing microfluidic-electrospray is on the order of ~10^2^ Hz (Fig. 3b and Fig. 5a,b). This suggests that the intrinsic pulsating frequency is closely related to the interfacial tension. Moreover, we found that the nozzle diameter *d*_*1*_, the flow rates, and the applied *E* also affect the frequency. One hypothesis is that the intrinsic pulsating frequency relates with the capillary wave on the charged liquid interface. To test our hypothesis, we have the following from the capillary wave equation^34, 35^:

1 $${f}^{2}=\frac{2}{{\pi }^{2}}\frac{\gamma }{\rho {r}^{3}}$$

Where *f* is the intrinsic pulsating frequency, *ρ* is the liquid density, and *r* is the radius of the emitted jet. The eq. (1) is supported by the interfacial tension data, where *γ*_*ow*_ ∼ 1–4 mN/m in our system, at least 18 times lower than *γ*_*w*_ ∼ 72 mN/m in electrospray-in-air. Furthermore, we vary different controlling parameters such as *d*_*1*_, *Q*_*1*_, *Q*_*2*_ and *E* to verify the scaling relationship between the radius of emitted jet, *r*, and the intrinsic frequency, *f*. Indeed, the data collapses onto a dash line on a log-log plot with a scaling exponent of −1.5, in consistence with eq. (1) (Fig. 5c). These experimental evidence supports the capillary wave explanation. Therefore, we can tune the average size of the resultant droplets through changing the intrinsic pulsating frequency, using approaches such as increasing electrostatic and shear stress to vary the emitted jet radius. The results described here are potentially important in fabricating sub-micron emulsions and particulate delivery systems.

## Discussion

Emulsion droplets of fine size are ubiquitous in our daily life, in industrial products, and are essential for emerging photo-acoustic therapies. Current emulsification either generates nano-sized emulsions with litter control over the characteristics of droplets, or precisely-controlled beautifully-uniform droplets that are too large for therapeutic purposes. We explored the microfluidic-electrospray technique by characterizing its operation modes and underlying mechanism. We found that a low-frequency pulsating mode, where the charge and fluid transportation are unbalanced, is responsible for droplets with a broad size distribution. By adjusting the imposing flow rate, an intrinsic high-frequency pulsating is pronounced, and through which the average droplet size can be manipulated. Our results could be beneficial to emulsion-based applications including food, cosmetics and pharmaceutics.

## Methods

### Fabrication of capillary microfluidic device

We fabricated capillary microfluidic device by aligning two tapered round capillaries into a square one^16, 17, 19–23, 28, 33^ (Fig. 1a). The inner and outer diameter of the round capillaries are 0.58 mm and 1 mm respectively; those of the square one are 1 mm and 1.05 mm, respectively. The tip of the round capillaries was tapered to sizes between 20 µm and 580 µm. One or multiple liquids were injected using syringe pumps (Longer Pump) and soft micro-tubings (Scientific Commodities). The inner phase flowed through the injection capillary (Fig. 1a); an outer liquid phase flowed via the gap between the injection and square capillaries, then into the collection capillary. The outer liquid phase completely wetted the glass wall that was hydrophobitized by trimethoxysilane (Sigma).

### Integration of the electrospray in a capillary microfluidic device

A metal wire was inserted into the injection capillary, and it was connected to the positive electrode of a direct current (DC) high voltage power supply. The device was grounded by connecting a metal tube wrapping the collection capillary to the negative end of the power supply. The direction of the electric field generated was the same as the flow direction of the inner liquid. The injected liquid phase was charged through the injection nozzle. The applied electric field intensity was controlled by adjusting the potential value of the power supply, *U*, while the distance between electrodes were kept constant. The applied electric field intensity typically ranged from 0 kV/cm to 6 kV/cm. The pulsating modes of the charged liquid jets were visualized and recorded using a high speed camera (Phantom M110) coupled with an inverted microscope (Motic AE2000).

### Composition of the liquid phases

The inner liquid was deionized water; the outer liquids were dielectric oils, including paraffin, silicone oil or squalene, with viscosities of 40 mPa.s, 10 mPa.s and 27.8 mPa.s respectively. The electrical conductivity of the liquid affects the operation modes that could occur for charged liquid meniscus. We measured the conductivity of the water phase in our experiment to be 2.34 µS/cm, which can be classified into leaky dielectrics; while the electrical conductivity of the oil phases is on the order of 10^−9^ µS/cm, thus the potential was mainly fall on the water phase. To tune the interfacial tensions, we added surfactants, such as span 80 (Sigma) or ABIL EM 90 (Evonik) to the outer phase. The concentration of span 80 and ABIL EM 90 were both 5 wt% that are higher than their critical micelle concentrations. The interfacial tension between paraffin, silicone oil, squalene with high concentration of surfactants and water, respectively, were measured to 3.1 mN/m, 2.8 mN/m, 2.5 mN/m. Due to the small size (0.02~1 mm) of the device, the corresponding Reynolds number was on the order of 10^−3^ or below, thus we neglected the inertia of the inner phase.

### Image analyzing

The pulsating frequency, jet radius and tip length of meniscus were obtained after processing and analyzing the high-speed images and videos using an open-source image-processing software, Image J (version: 1.48 v). The frame rates of the camera used for experiments were 400 frames·s^−1^ and 1000 frames·s^−1^. We counted the frames during the pulsating cycles and calculated the corresponding frequency. Each frequency was based on measurements from at least five different cycles while each jet radius was measured using at least five high-speed images.

## Electronic supplementary material

Supplementary Video S1

Supplementary Video S2
